# Anomaly Detection in Satellite Telemetry Data Using a Sparse Feature-Based Method

**DOI:** 10.3390/s22176358

**Published:** 2022-08-24

**Authors:** Jiahui He, Zhijun Cheng, Bo Guo

**Affiliations:** College of Systems Engineering, National University of Defense Technology, Changsha 410073, China

**Keywords:** anomaly detection, telemetry data, sparse features, OCSVM, time series

## Abstract

Anomaly detection based on telemetry data is a major issue in satellite health monitoring which can identify unusual or unexpected events, helping to avoid serious accidents and ensure the safety and reliability of operations. In recent years, sparse representation techniques have received an increasing amount of interest in anomaly detection, although its applications in satellites are still being explored. In this paper, a novel sparse feature-based anomaly detection method (SFAD) is proposed to identify hybrid anomalies in telemetry. First, a telemetry data dictionary and the corresponding sparse matrix are obtained through K-means Singular Value Decomposition (K-SVD) algorithms, then sparse features are defined from the sparse matrix containing the local dynamics and co-occurrence relations in the multivariate telemetry time series. Finally, lower-dimensional sparse features vectors are input to a one-class support vector machine (OCSVM) to detect anomalies in telemetry. Case analysis based on satellite antenna telemetry data shows that the detection precision, F1-score and FPR of the proposed method are improved compared with other existing multivariate anomaly detection methods, illustrating the good effectiveness of this method.

## 1. Introduction

Due to the rising complexity of spacecraft systems, in-orbit failures happen frequently. Before the 1990s, the in-orbit failure rate of satellites was 0.29 in China; however, these data rose to 1.12 in the early 2000s [1], seriously threatening the reliability and safety of spacecraft. Anomaly detection, which is the most efficient method of identifying unusual or unexpected events in orbit, is becoming a major issue in aerospace [2]. Spacecraft telemetry data are the key basis on which in-orbit spacecraft states are judged and anomalies on the ground detected. However, telemetry data are characterized by large data volume, high dimensionality, and complex relations, which creates severe challenges in realizing an anomaly detection method with a high detection rate, low false-positive detection rate, and strong interpretability [3].

Data-driven methods undertaken in the area of spacecraft anomaly detection in recent years [4] can generally be classified into two subcategories, namely, error-based and similarity-based methods.

In error-based methods, a reconstruction model is established to reconstruct the telemetry sequence based on training data, and anomalies are identified if the reconstruction errors exceed a given threshold. The advantage of error-based methods is their high detection efficiency. The disadvantage is that an accurate reconstruction model is difficult to establish and the threshold is difficult to select, requiring professional knowledge. Prediction models have been intensively developed for spacecraft anomaly detection thanks to their good performance in sequence reconstruction, and include support vector machine (SVR) [5,6], extreme learning machine (ELM) [7,8], and long short-term memory (LSTM) [9,10,11,12,13]. More recently, novel error-based methods based on sparse representation (SR) have been proposed for spacecraft anomaly detection. Pilastre et al. [14] decomposed telemetry signals into a dictionary using SR and analyzed the residuals resulting from this sparse decomposition to detect potential anomalies, however, this method cannot deal with correlation anomalies between continuous parameters. Takeishi et al. [15] extended SR using Singular Value Decomposition (SVD) to reconstruct the sparse matrix in order to detect correlation anomalies in multivariate time series by analyzing the reconstruction residuals. However, it is difficult to select the number of the retained singular values, which seriously affects detection performance.

Similarity-based methods consider telemetry data as a combination of sample points and identify the samples with low similarity as abnormal data from the sample set; these methods include clustering-based models [16,17] such as K-means [18] and fuzzy C-means (FCM) models [19] as well as classification-based models. The benefit of similarity-based methods is that they relax assumptions about data distribution and prior knowledge, and can mine relationships between telemetry data. In particular, clustering-based models do not need labeled data. However, it is difficult to determine the appropriate measurement of similarity. By contrast, classification-based models are more effective because they have strong learning ability and do not require similarity measurement. Considering the lack of abnormal samples in telemetry data, one-class classifiers such as OCSVM represent a more suitable choice for conducting anomaly detection in spacecraft. Hu et al. [20] defined six meta-features as inputs to OCSVM and built an anomaly detection model for use with time series. Saari et al. [21] selected frequency-domain features as inputs to OCSVM for detecting anomalies in wind turbine bearings. Vos et al. [22] combined LSTM and OCSVM for gearbox anomaly detection, using the LSTM prediction error as a feature and OCSVM to perform the detection task. One-class classifiers can solve the anomaly detection problem well, although because of the high dimensionality of telemetry data feature extraction and selection need to be carried out before building the one-class classifier.

Sparse representation has been shown to perform efficiently in local dynamics feature extraction of time series, and has been combined with the multivariate detection rule or SVD model in [14,15] in novel attempts. However, these methods have limited learning ability, only conditional constraints, and poor generalization performance. Considering this, SR combined with OCSVM is a more suitable choice for conducting anomaly detection in spacecraft.

For all of the reasons above, this paper proposes SFAD, a novel spacecraft anomaly detection method based on sparse features which can accurately identify hybrid anomalies from in-orbit telemetry data. The main contributions of this paper include:

1. A novel spacecraft anomaly detection method named SFAD; the proposed SFAD has the advantage of handling multivariate telemetry time series, and in particular is able to consider correlation anomalies between different telemetry parameters. To the best of our knowledge, few studies have been conducted to investigate sparse representation one-class classification in the context of satellite anomaly detection; our paper thus provides a reference for further study.
2. The proposed SFAD method is applied to achieve hybrid anomaly detection from satellite telemetry data, demonstrating its effectiveness and superiority over the standard OCSVM, LSTM-based, and SR-based methods for tackling the challenges related to spacecraft anomaly detection.

The remainder of this paper is organized as follows: Section 2 introduces the characteristics of telemetry data and anomalies; in Section 3, the proposed SFAD method is presented; Section 4 introduces the process of sparse feature extraction, including sparse representation, dictionary learning theories, sparse model construction for telemetry data, and the definition of sparse features; Section 5 introduces the OCSVM detection model based on sparse features; Section 6 describes an example satellite anomaly detection problem that is used to evaluate the performance of the proposed method; finally, our conclusions are drawn in the last section.

## 2. Analysis of Spacecraft Anomalies

### 2.1. Characteristics of Telemetry Data

Spacecraft telemetry data consist of hundreds to thousands of parameters; some are digital (e.g., switching status), and others are analogue (e.g., temperature, current). Due to the environment of space, telemetry data have certain typical characteristics. The primary points are as follows.

(1) Large data volume, high dimensionality and complex relationship between parameters: Spacecraft are highly precise and highly complex devices. To monitor their in-orbit status in real time, the transmission interval is typically milliseconds, and the satellite life is long, resulting in a large amount of data. In addition, there are complex structural relations between telemetry parameters.
(2) Complex temporal characteristics: Affected by the working mechanism and degradation of the spacecraft, certain telemetry parameters change periodically or exhibit certain trends. Different working modes lead to typical dynamics in parameters, which are called patterns. With certain types of random noise, telemetry anomalies are more difficult to identify.
(3) Many missing points and outliers: The influence of the environment of space and transmission distortion can both lead to missing points and outliers in telemetry parameters; therefore, proper pre-processing is essential. For example, the box-plot method, 53H method, and median filtering algorithm are classical methods that remove outliers caused by errors in data conversion and transmission. Several different reconstruction methods have been applied to compensate for missing points [23].

### 2.2. Anomalies in Telemetry

Telemetry data are collected at a specific sampling frequency, and are thus presented in the form of a time series. The types of anomalies in the telemetry are similar to the time series, and can be divided into point anomalies, collective anomalies, contextual anomalies, and correlation anomalies [14], which are summarized below.

**Figure 1:**
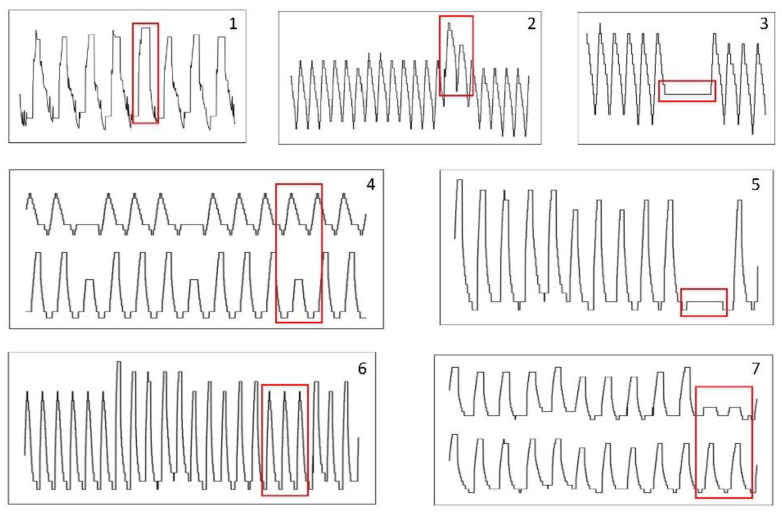
Examples of anomalies (highlighted in red boxes) considered in this work.

(1) Point anomalies are single sample points or small continuous sample points that are markedly different from the rest of the data. An example of a point anomaly is shown in Figure 1 (box #2).
(2) Collective anomalies are single points that do not exceed the threshold, but which make up a collection of continuous points that violates the regularity of the original data. We consider a collection of continuous points as collective anomalies. Three examples of collective anomalies are shown in Figure 1 (boxes #1, #3 and #5).
(3) Contextual anomalies occur when a point is abnormal only in a particular context. In most cases, the contextual environment of telemetry data is time; thus, contextual anomalies in this study can be understood to be contextual anomalies of time. An example of a contextual anomaly is shown in Figure 1 (box #6).
(4) Correlation anomalies occur when the correlation between parameters changes. Such correlations include physical relations, logical relations, pattern relations, etc. Figure 1 (boxes #4 and #7) shows examples of correlation anomalies. The anomalies in Figure 1, boxes #4 and #7 consist of relationships of time-series patterns that have changed. In the examples, the expected co-occurrence patterns of parameters are not observed in the red box, which corresponds to a correlation anomaly.

The detection of collective anomalies and certain contextual anomalies requires observing the parameter behaviors in a certain time window in order to extract relevant time-domain characteristics. For correlation anomalies, the multivariate framework must be considered to extract the time-domain and correlation characteristics among parameters. The objective of this study is to propose a flexible multivariate anomaly detection method that is sensitive to hybrid anomalies in telemetry.

## 3. Sparse Feature-Based Anomaly Detection Method (SFAD)

**Figure 2:**
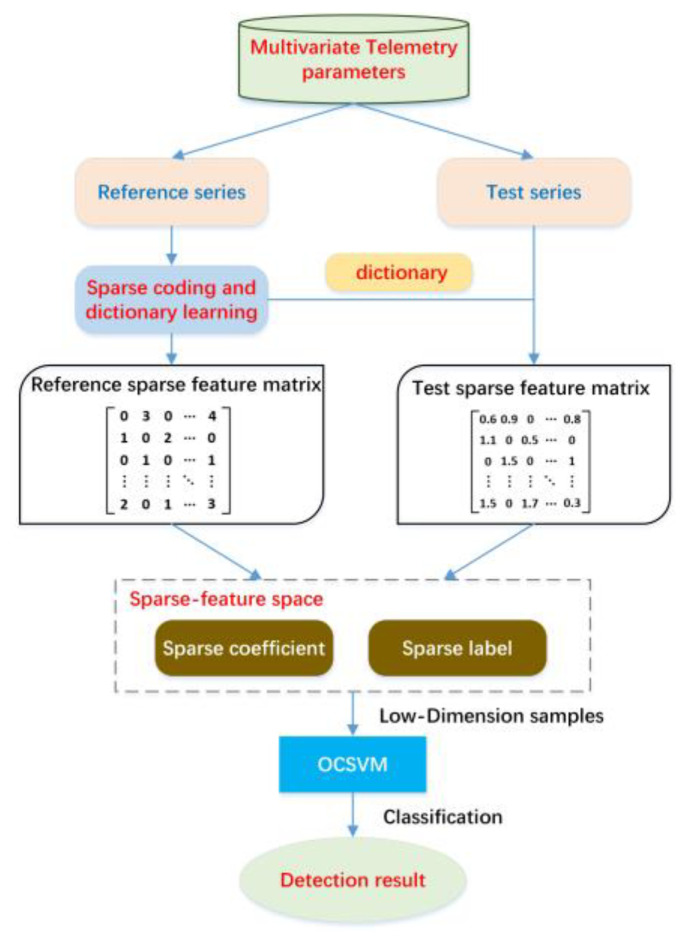
Flowchart of the SFAD method for spacecraft anomaly detection.

**Figure 3:**
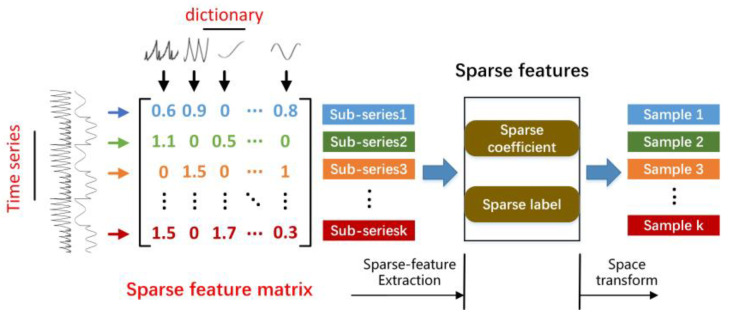
Semantic graph of sparse feature space.

The SFAD method shown in Figure 2, can be divided into two parts, offline and online. In the offline part, dictionary learning algorithms are used to capture normal patterns (i.e., atoms) from the reference spacecraft’s multivariate telemetry data while producing a reference sparse feature matrix describing the local dynamics and co-occurrence relations of telemetry parameters. Then, defined sparse features (i.e., sparse coefficients and sparse labels) are extracted from the sparse feature matrix to reduce the feature dimension, as shown in Figure 3. In the online part, the online monitoring of the telemetry parameter data can be sparsely coded with the dictionary learned from reference data that do not contain any anomalies. Similarly, the online sparse matrix is transformed into a sparse feature space. Finally, we identify anomalies using OCSVM on the sparse feature space. According to the above description, sparse features drastically reduce the size of the multidimensional sequences in a sliding window while retaining the local dynamics and co-occurrence relations. By feeding lower-dimensional samples to the OCSVM, the accuracy and efficiency of detection can be markedly improved, which is suitable for online detection.

## 4. Sparse Feature Extraction

### 4.1. Sparse Representations and Dictionary Learning

Sparse representations have received widespread attention in signal and image processing applications, including signal denoising [24], detection of abnormal video motions [25] and irregular heartbeat detection [26]. The basic theories are described below.

When building a sparse problem, a signal (i.e., a vector) $y\;\in \;R^{W}$ is equivalent to y ≈ Dx, where $D\;\in \;R^{W\times M}$ is a dictionary composed of M columns called atoms and $x\in R^{M}$ is a sparse vector. Thus, the signal y can be linearly represented by a few atoms in D. The sparse problem is formulated as follows:

(1) $$\begin{array}{c} \hat{x}=\arg\underset{x}{\min}{\left\|y-Dx\right\|}_{2}^{2}\\ \begin{array}{c} s.t.\;\begin{array}{c} {\left\|x\right\|}_{0}\leq T_{0} \end{array} \end{array} \end{array}$$

where $∥\;{∥}_{0}$ is the $l_{0}$ pseudo-norm, which counts the number of nonzero entries in x; $∥\;{∥}_{2}$ is the $l_{2}$ norm; and $T_{0}$ is the maximum of nonzero entries in x.

Problem (1) is NP-hard, and the common solutions include greedy algorithms such as matching pursuit (MP) [27] and orthogonal matching pursuit (OMP) [28], or constrained optimization algorithms such as the alternating direction multiplier method (ADMM) [29].The performance of the sparse representation y ≈ Dx strongly relies on the choice of dictionary D. Dictionaries can be divided into two categories: parametric dictionaries composed of fixed atoms, such as wavelet or discrete cosine transform (DCT), and data-driven dictionaries, in which atoms are learned from data. The latter has resulted in more attention for practical applications. The dictionary learning problem is formulated as follows:

(2) $$\begin{array}{l} \hat{x},\hat{D}=\arg\underset{x,D}{\min}{\left\|y-Dx\right\|}_{F}^{2}\\ \begin{array}{ccc} s.t. & & {\left\|x\right\|}_{0} \end{array}\leq T_{0} \end{array}$$

Classical dictionary learning algorithms typically consist of two steps: sparse coding and dictionary updating. These steps then update alternately, such as in K-SVD [30].

### 4.2. Sparse Model Construction for Telemetry Data

#### 4.2.1. Preprocessing

**Figure 4:**
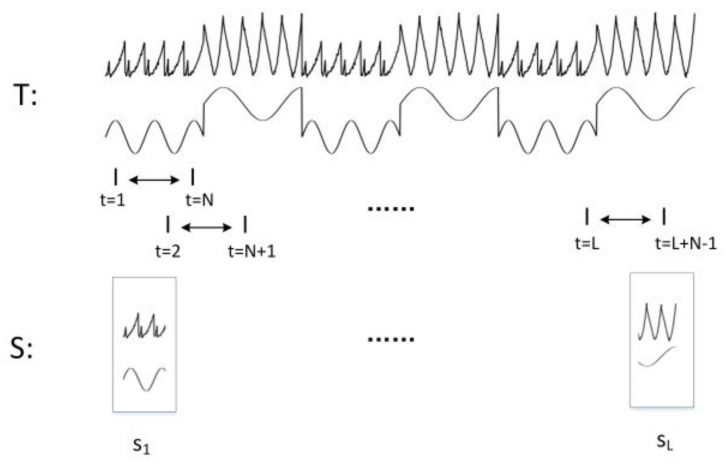
Segmentation of telemetry into sliding windows.

The multivariate anomaly detection framework requires that we cannot consider sample points separately, and must consider time-domain and correlation characteristics over a duration. This study uses a sliding window technique to segment multidimensional sequences in telemetry, as shown in Figure 4. Telemetry time series (i.e., $T={\left[y_{1},\;\;y_{2},\cdots ,\;y_{K}\right]}^{T}$, where K is the number of telemetry parameters) are assumed to be segmented into windows with a fixed size N with a shift of 1 and an overlapping area equal to N−1. Each window matrix is concatenated into vectors as a sample vector $s_{i}$, i=1, 2,⋯, L. Then, sample vectors are concatenated by time step as a sample matrix S, where $S\;\in \;R^{KN\times L}$.

#### 4.2.2. Sparse Representation for Telemetry Data

In the offline part, considering preprocessing, input reference telemetry data were converted to the sample matrix $S_{train}={\left[s_{train}^{\left(1\right)},\;s_{train}^{\left(2\right)},\cdots ,\;s_{train}^{\left(K\right)}\right]}^{T},s_{train}^{\left(i\right)}\;\in \;R^{N\times L_{1}}$. Dictionary learning for each parameter sample metric $s_{train}^{\left(i\right)}$ was used to capture the normal patterns in telemetry, with the corresponding sparse matrix $X_{train}^{\left(i\right)}$ and dictionary $D_{i}$ obtained by the following optimization:

(3) $$\begin{array}{l} X_{train}^{\left(i\right)},D_{i}=\arg\underset{X,D}{\min}{\left\|s_{train}^{\left(i\right)}-D_{i}X_{train}^{\left(i\right)}\right\|}_{F}^{2}\\ \;\;\;\;\;\;\;\;\;\;\;\;\;\;\;\;s.t.\;\;\;\;{\left\|x_{j}\right\|}_{0}\leq T_{0} \end{array}$$

We solve problem (3) iteratively, optimizing $X_{train}^{\left(i\right)}$ with $D_{i}$ fixed and vice versa. We then use the K-SVD algorithm; the detailed steps of the K-SVD are described in Algorithm 1.

**Algorithm 1:** K-SVD**input: sample** $s_{train}^{\left(i\right)}$, **dictionary** $D_{i}$, **sparse matric** $X_{train}^{\left(i\right)}$**output: dictionary** $D_{i}$, **sparse matric** $X_{train}^{\left(i\right)}$**1. initialization: set the initial dictionary** $D_{i}^{\left(0\right)}\in R^{N\times M}$ **with** $l_{2}$ **normalized columns, set** j=0 **2. sparse coding: solve the sparse matric** $X_{train}^{\left(i\right)\left(j\right)}\in R^{M\times L_{1}}$ **with** $D_{i}^{\left(j\right)}$ **3. dictionary update: for each column** $d_{k},k=1,2,\cdots ,M$ **in** $D_{i}^{\left(j\right)}$, **update it by** **----define the group of examples that use this atom,** $w_{k}=\left\{m|1\leq m\leq L_{1},\;x_{T}^{k}\left(m\right)\neq 0\right\}$, $x_{T}^{k}$ is the *k*-th row in $X_{train}^{\left(i\right)\left(j\right)}$.
**----compute the overall representation error matrix** $E_{k}$ **by** $E_{k}=s_{train}^{\left(i\right)}-\underset{z\neq k}{\sum }d_{z}x_{T}^{z}$
**----restrict** $E_{k}$ **by choosing only the columns corresponding to** $w_{k}$**, and obtain** $E_{k}^{R}$
**----apply SVD decomposition** $E_{k}^{R\;}=U\Sigma V^{T}$**. Choose the updated dictionary column** $\tilde{d_{k}}$ **to be the first column of** U**. Update the coefficient vector** $x_{k}^{R}$ **to be the first column of** V
**multiplied by** $\Sigma \left(1,1\right)$
**4. set** j=j+1

For each telemetry parameter $y_{i}\;=\;\left(1,\;2,\cdots ,\;K\right)$, we have the approximate expression of $s_{train}^{\left(i\right)}\;=\;D_{i}X_{train}^{\left(i\right)}$ by K-SVD; then, the sparse representation algorithm in K-SVD chooses OMP. The entire reference sample matrix $S_{train}=\;\;{\left[s_{train}^{\left(1\right)},\;s_{train}^{\left(2\right)},\cdots ,s_{train}^{\left(K\right)}\right]}^{T}$ can be written in matrix form as follows:

(4) $$\begin{array}{ll} S_{train} & =DX_{train}\\ & =\left[\begin{array}{cccc} D_{1} & 0 & \cdots & 0\\ 0 & D_{2} & \cdots & 0\\ \vdots & \vdots & \ddots & \vdots \\ 0 & 0 & \cdots & D_{K} \end{array}\right]\cdot \left[\begin{array}{c} X_{train}^{\left(1\right)}\\ X_{train}^{\left(2\right)}\\ \vdots \\ X_{train}^{\left(K\right)} \end{array}\right] \end{array}$$

The learning dictionary $D_{i}$ consists of elemental dynamics of the reference time series $y_{i}$ within a given time scale according to the size of the sliding window N. Considering the sparse coding, the sparse matrix is a set of coefficients of the bases and compresses the information on the dynamics and relations of the multivariable sequence. In the online part, we first used preprocessed real-time telemetry data to obtain the test sample matrix $S_{test}={\left[s_{test}^{\left(1\right)},s_{test}^{\left(2\right)},\cdots ,s_{test}^{\left(K\right)}\right]}^{T}$; only sparse representations $X_{test}^{\left(i\right)}$ are optimized by the OMP algorithm with the fixed learning dictionary $D_{i}$ for each parameter in telemetry. Similar to the reference sample matrix $S_{train}$, the test sample matrix has the same matric form as follows:

(5) $$\begin{array}{ll} S_{test} & =DX_{test}\\ & =\left[\begin{array}{cccc} D_{1} & 0 & \cdots & 0\\ 0 & D_{2} & \cdots & 0\\ \vdots & \vdots & \ddots & \vdots \\ 0 & 0 & \cdots & D_{K} \end{array}\right]\cdot \left[\begin{array}{c} X_{test}^{\left(1\right)}\\ X_{test}^{\left(2\right)}\\ \vdots \\ X_{test}^{\left(K\right)} \end{array}\right] \end{array}$$

#### 4.2.3. Definition of Sparse Features

**Figure 5:**
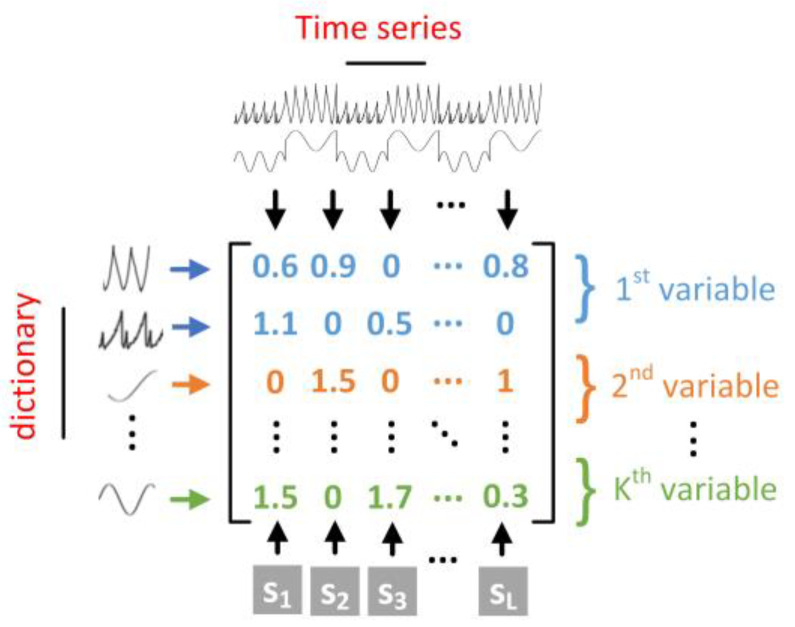
Conceptual diagram of the sparse matrix.

As described in the introduction above, the dynamics and co-occurrence relations in the multivariate telemetry data are extracted into a sparse matrix. However, the sparse matrix contains a great deal of useless information; for example, we typically choose a dictionary of hundreds of atoms to ensure the accuracy of sparse representation and set the maximum of nonzero entries $T_{0}$ to a small value, resulting in most of the zero elements in sparse matrices. To markedly reduce the dimensionality of the sparse matrix and provide a more appropriate input for the classification-based anomaly detection approach in the latter part, we must capture the key elements. In the sparse matrix, the columns correspond to the telemetry parameter, the rows correspond to local patterns, and each element denotes the weight of the elemental patterns, as shown in Figure 5. A sliding window is represented by a column vector in the sparse matrix; thus, we consider the numerical order and coefficient of nonzero elements in order to determine the local dynamic behaviors of sequences in sliding windows. For each column vector in the sparse matrix, we define two types of sparse features:

(1) The sparse label records the numerical order of the nonzero elements, equal to the row number of the element, and concatenates it in order into a sparse label vector.
(2) The sparse coefficient records the coefficient of the nonzero elements and concatenates it in order into a sparse coefficient vector.

The size of the sparse features is related to the maximum constraint of $T_{0}$ and the number of telemetry parameters K, and is a $2T_{0}K-D$ sample. The local patterns relative to the entire sliding window and co-occurrence relations of patterns among parameters are transformed into a $2T_{0}K-D$ sparse-features space, which is a low-dimensional space suitable for an efficient and accurate classifier. Thus, the reference sparse matrix $X_{train}\;\in \;R^{KM\times L_{1}}$ and the test sparse matrix $X_{test}\;\in \;R^{KM\times L_{2}}$ are transformed into the reference sparse feature matrix $F_{train}\;\in \;R^{2T_{0}K\times L_{1}}$ and test sparse feature matrix $F_{test}\;\in \;R^{2T_{0}K\times L_{2}}$.

## 5. Sparse Feature-Based Anomaly Detection Using OCSVM

**Figure 6:**
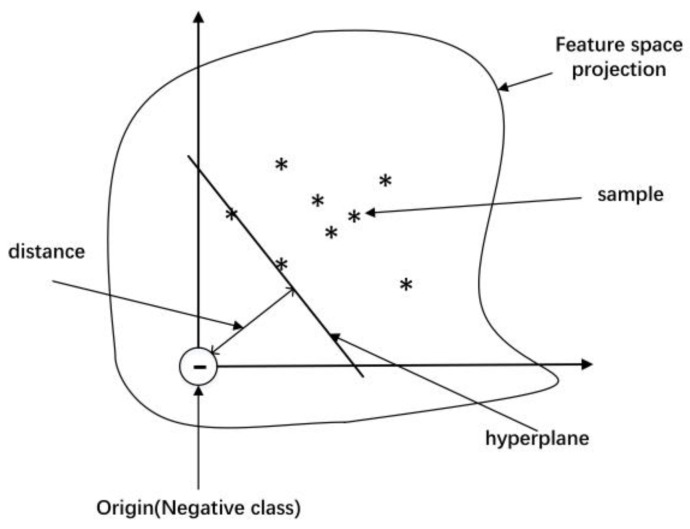
Schematic of the OCSVM.

One-class support vector machines were first proposed by Schölkopf to solve the single classification task [31]. As shown in Figure 6, the essential concept of the OCSVM algorithm is to map the samples in a higher-dimensional subspace and then to find a hyperplane in this subspace separating the one-class samples from the origin, which in this case involves maximizing the distance from the hyperplane to the origin while separating most of the one-class samples. For a new sample, if it is over the hyperplane (i.e., away from the origin) it is classified as the positive class, and vice versa.

The OCSVM is advantageous because it achieves high efficiency in training and testing and requires few hyperparameters from the user. More importantly, the OCSVM eliminates the need for labelled two-class information during training, which is suitable for telemetry data because the anomalies in telemetry are rare.

Considering the reference sparse-feature matrix $F_{test}\;\in \;R^{2T_{0}K\times L_{2}}$, the goal of the OCSVM is to map the reference samples in a higher-dimensional subspace H with a transformation φ and to find a hyperplane in this subspace, separating the one-class samples from the origin. The hyperplane is found by solving the following problem:

(6) $$\begin{array}{c} \min\frac{1}{2}{\left\|w\right\|}^{2}\\ s.t.\;\;\;\;w\varphi \left(F_{train}(:,j)\right)+b\geq 0,j=1,2,\cdots ,L \end{array}$$

where ω and b are parameters of the hyperplane. However, we cannot absolutely separate the one-class samples from the origin; thus, we add the slack variable $\xi_{i}$ and a relaxation factor v to Equation (6):

(7) $$\begin{array}{c} \min\frac{1}{2}{\left\|w\right\|}^{2}+\frac{1}{vL}\sum_{j=1}^{L}\xi_{j}+b\\ \begin{array}{cc} s.t. & w\varphi \left(F_{train}(:,j)\right) \end{array}+b+\xi_{j}\geq 0,\xi_{j}\geq 0,\left(j=1,2,\cdots ,L\right) \end{array}$$

Equation (7) is a convex quadratic programming problem, and we solve it by introducing the Lagrange function:

(8) $$\begin{array}{c} \min\alpha_{i}\alpha_{j}K\left(F_{train}\left(:,i\right),F_{train}\left(:,j\right)\right)\\ s.t.\;\;\;\;0\leq \alpha_{i}\leq 1,\sum_{i=1}^{L}\alpha_{i}=vL \end{array}$$

where $K\left(\cdot \right)$ is the kernel function. The derivation process of Equation (8) refers to [31]. The decision function of the classifier can be formulated as follows:

(9) $$\begin{array}{ll} f\left(y\right) & =sign\left[w\varphi \left(F_{train}\left(:,i\right)\right)+b\right]\\ & =sign\left[\sum_{i=1}^{L}\alpha_{i}K\left(F_{train}\left(:,i\right),F_{train}\left(:,j\right)\right)+b\right] \end{array}$$

where the sign is the function defined by

(10) $$sign\left(y\right)=\left\{\begin{array}{cc} 1 & if\;y\geq 0\\ -1 & if\;y<0 \end{array}\right.$$

In this paper, we define an anomaly score as the distance between the test sparse feature vector y and the hyperplane:

(11) $$anomaly\;score=\left\{\begin{array}{cc} \frac{-w\varphi \left(y\right)-b}{\left\|w\right\|} & if\begin{array}{c} f\left(y\right)<0 \end{array}\\ 0 & if\begin{array}{c} f\left(y\right)\geq 0 \end{array} \end{array}\right.$$

## 6. Illustrative Cases

### 6.1. Case Settings

To verify the effectiveness of the proposed method, the telemetry data of fourteen parameters collected from a satellite antenna were investigated. The parameters recorded were the temperature, current, and on–off state of the satellite’s components. Before conducting the experiment, we selected the parameters based on elementary data analysis and expert experience, which allowed us to describe the abnormal states of the antenna. The selected parameters are affected by the positions of the satellite and the Sun; thus, the change rule is cyclical on an annual and daily scale. Considering the dispersion of the anomalies, we investigated seven nonoverlapping subseries from the original series that contained anomalies. The seven subseries are shown in Figure 1 and are represented as Anomaly 1–7 in the later text. Relevant metrics of the data are shown in Table 1.

During sparse features extraction, we applied the pre-processing described in Section 4.2.1 with the parameter N=100 and used the K-SVD algorithm for dictionary learning from reference telemetry data. For the K-SVD, we built a dictionary with M=1100 atoms (the choice of M is discussed later), and the number of iterations was set to 100 to ensure the minimum residuals of sparse coding. The sparse coding algorithm OMP is used in this study, and the sparse parameter $T_{0}=6$ was set (the choice of $T_{0}$ is discussed later). The Gaussian RBF kernel is employed in the OCSVM model, and the kernel parameters C and γ were optimized by grid search. In the grid search, parameters C and γ change from 0 to 1 with a step of 0.01.

Considering the imbalance of normal and anomaly data, the detection rate alone is insufficient to evaluate the performance of the anomaly detection approach. In this study, we consider four evaluation indicators: precision, recall, *F*1-score, and *FPR*. The definition of these metrics is described as follows:

(12) $$precision=\frac{TP}{TP+FP}$$

(13) $$recall=\frac{TP}{TP+FN}$$

(14) $$F1\text{-}score=\frac{2recall*precision}{recall+precision}=\frac{2TP}{2TP+FP+FN}$$

(15) $$FPR=\frac{FP}{TN+FP}$$

where *TP*, *FP*, *TN*, and *FN* denote the number of normal series correctly detected as normal (true positives), the number of abnormal series detected as normal (false positives), the number of abnormal series detected as abnormal (true negatives) and the number of normal series detected as abnormal (false negatives), respectively. The larger the precision, recall, and *F*1-*score* are, the better the model performance. The lower the *FPR* is, the better the model performance.

### 6.2. Experimental Results and Methods of Comparison

#### 6.2.1. Experimental Results

**Figure 7:**
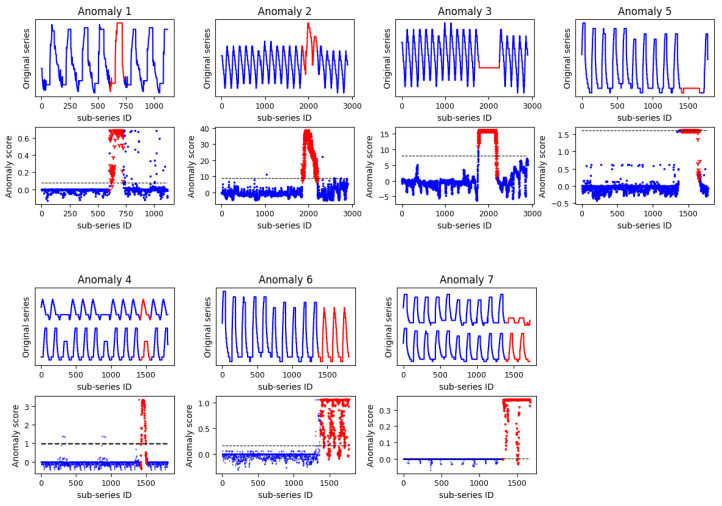
Anomaly scores for the seven subseries containing anomalies (for the original series, the blue solid lines denote the real normal series and the red lines denote the abnormal series; for the anomaly score, the blue solid dot denotes the real normal samples, the red * denotes the anomalies, the black dashed line denotes the anomaly detection threshold, and the subseries ID is the index of each point in the series).

This section applies the proposed method to satellite telemetry data and verifies the performance of the method. For each experimental subseries containing anomalies, we segmented the series to reference data (normal data) and test data (contains anomalies) in a proper proportion. Reference data were input into the K-SVD model for dictionary learning to extract the local dynamics and co-occurrence relations in a sparse matrix under normal conditions. The same operation was applied to the test data with the learned dictionary. Sparse feature vectors extracted from the sparse matrix were input to the OCSVM, and the anomaly score was output. The higher this score is, the higher the probability of anomalies. In this study, we set a threshold associated with the value of the pair (false-positive rate and true-positive rate) located closest to the ideal point (0,1) to avoid too many false alarms. Figure 7 shows the anomaly scores of seven experimental datasets, where red dots denote anomalies and the black dashed line denotes the detection threshold. It can be seen that the proposed method achieved superior performance with anomalies because most of the red dots exceeded the threshold. The proposed method thus exhibits wider adaptability to hybrid anomalies.

#### 6.2.2. Methods of Comparison

To test the effectiveness of the SFAD method in this paper, we experimentally compared two OCSVM-based methods and two latest anomaly detection methods in the field of anomaly detection. The details of the methods are described below.

The one-class support vector machine method (OCSVM): the OCSVM method was investigated in a multivariate framework using input vectors composed of telemetry parameters. The input vectors were obtained by preprocessing, as described in Section 4.2.1. Then, the OCSVM model was trained with reference input vectors and output the anomaly score of the test vectors [31].

The meta feature-based anomaly detection method (MFAD): Min Hu et al. [20] proposed an anomaly detection method for univariate or multivariate time series based on local dynamics. For each sliding window, the method first defines six meta features to statistically describe the local dynamic characteristics. The vectors composed of meta features were input to the OCSVM to complete training and anomaly identification. Multivariable time series were converted to new univariate time series by PCA technology in order for meta features to be extracted from sliding windows.

Anomaly detection using LSTMs and nonparametric dynamic thresholding (LSTM-NDT): Kyle et al. [9] proposed an anomaly detection method based on predicted models. Considering that LSTM achieves good performance in sequence modelling, LSTM was used to predict telemetry data. The residuals between the predicted and true values were considered based on the rule of anomaly identification. Then, a nonparametric dynamic thresholding approach was proposed to automatically determine the threshold without scarce labels or false parametric assumptions.

Anomaly detection with sparse representation (SRAD): Sparse representation has attracted increasing attention in the field of anomaly detection. Amir et al. [26] proposed that signals can be decomposed into this dictionary using SR, allowing potential anomalies to be detected by analyzing the residuals resulting from this sparse decomposition. Takeishi et al. [15] extracted the co-occurrence relations of local patterns from a sparse feature matrix by LSA and detected multivariate anomalies by analyzing the residuals resulting from SVD reconstruction. In this paper, we combine the advantages of these two methods; the first was applied for point, collective, and contextual anomalies, and the second for correlation anomalies.

**Figure 8:**
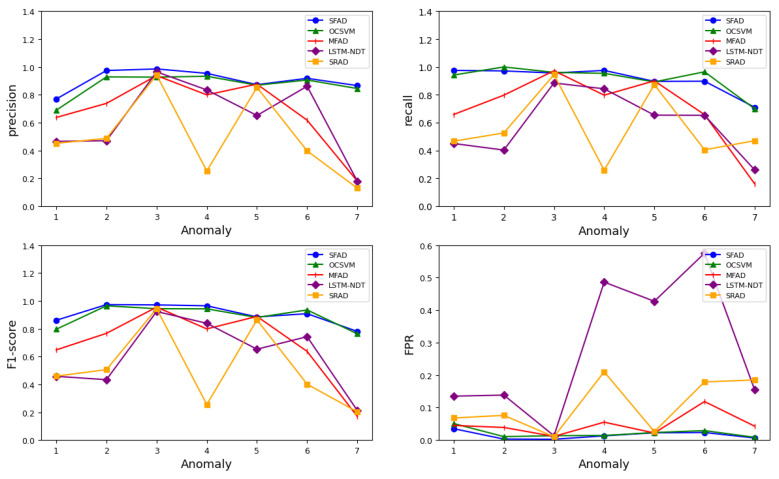
Evaluation indicator comparison of anomaly detection methods.

Table 2 and Table 3 show the evaluation effectiveness indicators for all methods of anomaly detection. The visualized results are shown in Figure 8. Comparing the indicators in Table 2 and Table 3 shows that the proposed SFAD method performs better than the other methods, primarily due to its higher precision, higher F1-score, and lower FPR. The latter is particularly important for spacecraft health monitoring, because too many false alarms are a problem for operational missions. In particular, the indicators are markedly improved in collective anomalies and correlation anomalies. From Figure 8, it can be seen that among the five methods the OCSVM-based methods are more accurate than the predicted method and sparse-based method. All of the lines of SFAD are stable, indicating better robustness and stability. In contrast, the other methods have limitations for certain abnormal datasets, leading to fluctuations in indicators.

### 6.3. Sensitivity Analysis

This section discusses the model parameters and dictionary learning methods, providing a reference for model optimization. We selected two key sparse model parameters and three types of dictionaries to choose the best model. The details are as follows.

#### 6.3.1. Selecting the Number of Atoms in the Dictionary

**Figure 9:**
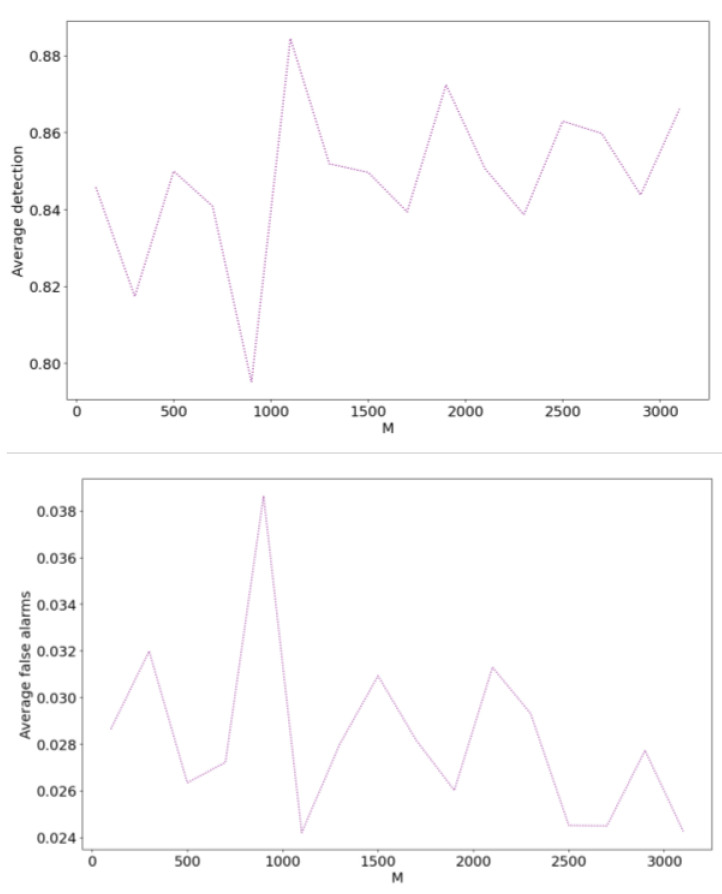
Values of average detection and average false alarms versus the number of dictionary atoms.

This section explains how the proposed SFAD method selects the number of atoms in the dictionary. Generally, the more atoms in the dictionary, the better the sparse representation of telemetry data and detection results. However, too many atoms can markedly increase computational complexity. Therefore, we set up an experiment to find the best number of atoms. We moved the number of atoms from 100 to 3100 with a step of 200, and used the average detection rate and average false alarms as the evaluation indicators. Figure 9 shows the indicator changes for different numbers of atoms.

As the number of atoms in the dictionary increases, indicators fluctuate and the general performance starts improving. For example, moving from 100 to 900 atoms, the average detection rate and average false alarms are 82.98% and 3.06% respectively. Beyond 1100 atoms, the corresponding indicators are 85.63% and 2.72% respectively, which is an improvement in general performance. It can be concluded that increasing the number of atoms leads to higher detection performance, however, this increase is not sustainable. Too many atoms in the dictionary may not improve the detection performance, instead wasting computing resources. Considering the balance between the detection effect and computing resources, we chose *M* = 1100 for this study. This analysis emphasizes that choosing the number of atoms in the dictionary is important for anomaly detection using SFAD.

#### 6.3.2. Selecting the Sparse Degree $T_{0}$

**Figure 10:**
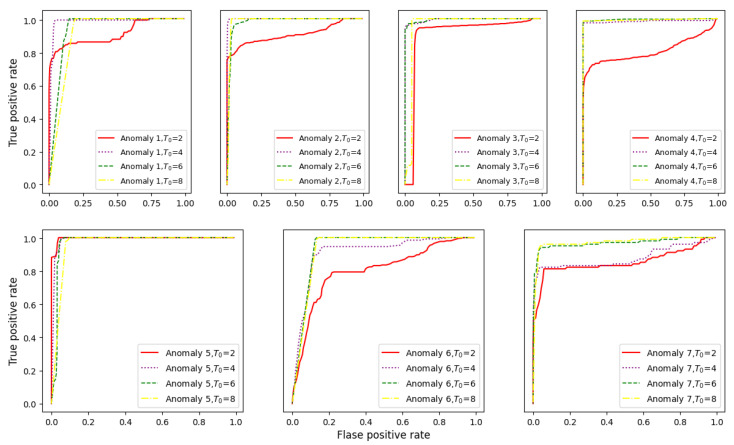
ROC curves for SFAD with different values of $T_{0}\;\in \;\left\{2,\;4,\;6,\;8\right\}$.

This section explains how the proposed SFAD method determines the sparse degree $T_{0}$ in the sparse coding algorithm. In the proposed method, the sparse degree affects the effect of sparse representation and determines the size of the sparse features; thus, considering the choice of the sparse degree $T_{0}$ is important for anomaly detection using SFAD. In an experiment, we set different sparse degree values ($T_{0}\;=\;\;2,\;4,\;6,\;8$) and determined the best value using ROCs to express the true positive rate as a function of the false positive rate. Figure 10 shows the ROCs with different $T_{0}$ for the seven anomaly datasets. The larger the area under the ROC curve is, the better the detection performance of the model. Among the four values, $T_{0}\;=\;2$ performs the worst; next, $T_{0}\;=\;4,\;6,\;8$ all achieve good performances effects on anomalies 1–5; however, $T_{0}=4$ is worse than $T_{0}=6,\;8$ on anomalies 6 and 7. Considering that $T_{0}\;=\;6$ leads to a good compromise in terms of performance and computational complexity (the higher $T_{0}$ is, the higher the computation time), we used $T_{0}=6$ with the sparse coding algorithm in this study. This analysis shows that a sparse degree that is too small results in poor performance in sparse representation and ignores necessary features as input to OCSVM, while an excessive sparse degree markedly adds to interference information during modelling and increases computational complexity. These results confirm that choosing a reasonable sparse degree in the sparse coding algorithm is important for anomaly detection using SFAD.

#### 6.3.3. Selecting the Dictionary

Considering the influence of different dictionaries on anomaly detection results, we applied data-driven dictionaries for sparse coding in the proposed method. In this experiment, we added parameter dictionaries for comparison, including the DCT dictionary and telemetry data dictionary (TD), which were initialized with M training samples selected randomly in the training database [14]. Only by changing the dictionary can we consider each of the KSVD-OMP-OCSVM method (the proposed method in this paper), DCT-OMP-OCSVM method, and TD-OMP-OCSVM method. The parameters in the models kept the initial settings from Section 6.1.

**Figure 11:**
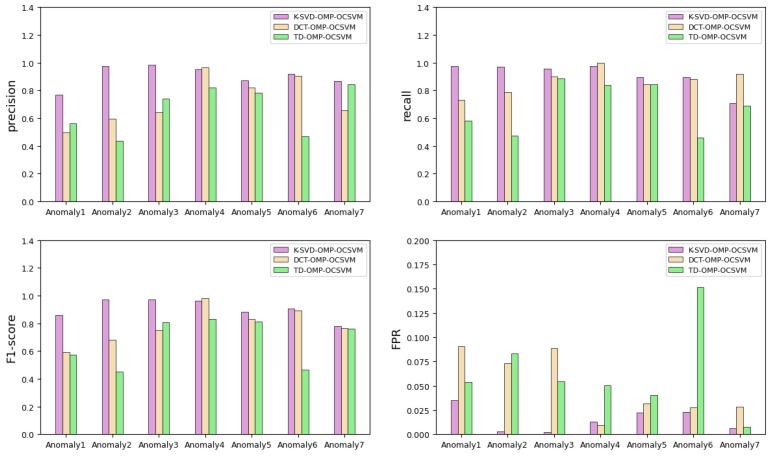
Comparison of evaluation indicators for different dictionaries in SFAD.

Table 4 shows the four evaluation indicators of the different dictionaries in SFAD. The optimal indicator value for each anomaly is shown in bold. In the different dictionaries, the K-SVD dictionary learning algorithm combined with OMP and OCSVM achieved the best performance in anomaly detection. The visualized results are shown in Figure 11. From the figure, it can be seen that the second-best algorithm is DCT-OMP-OCSVM, while TD-OMP-OCSVM performed worst. The K-SVD-OMP-OCSVM performs better and more stably in identifying hybrid anomalies. In contrast, TD-OMP-OCSVM has extreme cases when identifying certain anomalies, such as excessive FPR values in anomaly 6. The FPR score of K-SVD-OMP-OCSVM is markedly lower than those of the other methods. A lower false result rate is more suitable for online anomaly detection of telemetry data. Therefore, a data-driven dictionary is more applicable to the proposed approach and can more accurately describe the real characteristics of the data. In contrast, the DCT dictionary lacks connection to the data, and the TD dictionary describes characteristics incomprehensively, limiting their effects in SFAD.

### 6.4. Experiments Using Public Datasets

In order to verify the applicability of the SFAD method proposed in this paper, the public expert-labeled telemetry data derived from Incident Surprise Anomaly (ISA) reports for the Mars Science Laboratory (MSL) rover Curiosity were investigated. The validation data from MSL consisted of 27 telemetry channels, and one telemetry parameter and 54 telemetry commands were selected in a telemetry channel. The detailed information of the MSL dataset is shown in Table 5.

For simplicity, we regard anomaly sequences as the detection unit and set the detection rules as follows. Suppose that the interval of an anomaly sequence is $\left(t_{d},t_{u}\right)$, and the detected anomaly interval is $\left({\tilde{t}}_{d},{\tilde{t}}_{u}\right)$. First, a true positive is recorded if $num\left(\left(t_{d}-N,t_{u}\right)\cap \left({\tilde{t}}_{d},{\tilde{t}}_{u}\right)\right)\;\geq \;0.2num\left(\left(t_{d},t_{u}\right)\right)$, where num is the number of elements in the set. Second, a false negative is recorded if no detected anomaly intervals overlap with a real anomaly interval. Third, a false positive is recorded if no real anomaly intervals overlap with a detected anomaly interval. Precision, recall, and F1-score are selected for the corresponding performance indicators.

It worth noting that in MSL datasets there are two types of anomalies, point anomalies and contextual anomalies, which can occur in different telemetry channels, and their abnormal behaviors are different, which is a challenge when applying methods. We used the methods proposed in this paper compared to other methods of detecting anomalies; the obtained results are shown in Table 6.

It can be seen from Table 6 that, first, compared with other OCSVM-based methods, the precision, recall, and F1-score of SFAD are significantly better, which illustrates the better effectiveness of sparse features compared to statistical features in MFAD and no feature extraction in OCSVM. Second, when compared with other methods, SRAD performs worst, which illustrates that anomaly detection based on residuals resulting from sparse decomposition has limited learning ability, conditional constraint, and poor generalization performance. SR combined with OCSVM is a more suitable choice. LSTM-NDT detects the most point anomalies and contextual anomalies, which illustrates the good performance of prediction-based methods for univariate anomalies. In general, our experiments on public datasets illustrate the wide applicability of the SFAD proposed in this paper.

## 7. Conclusions

This paper proposes a novel anomaly detection method named SFAD for the detection of anomalous in-orbit telemetry data. The SFAD method is suitable for both univariate and multivariate time series as well as for online adaptive detection, as all previous subseries must only be calculated once. Case analysis shows that when compared with existing methods the proposed method achieves marked improvements in anomaly detection performance. The experiment in this study considers the influence of different dictionaries, the number of dictionaries, and the degree of sparsity. These experiments ensure the accuracy of the model and provide a reference for future extended application of the proposed anomaly detection framework.

In future research, the following issues should be investigated. First, we plan to improve the accuracy of detection by refining the sparse features to better summarize the local dynamics and correlations of time series and by applying extended models of the OCSVM. In addition, we plan to investigate the potential use of sparse coding to identify the causes of anomalies. These related research directions should help to develop online anomaly detection with high-dimensional data, which could be helpful for spacecraft health monitoring.

## Figures and Tables

**Table 1:** Description of data.

Attributes	Values
Number of telemetry parameters	14
Data sampling duration	366 days
Data sampling frequency	10 min
Length of each parameter	52,704
Number of univariate anomalies	5
Number of multivariate anomalies	2
Number of experimental datasets	7

**Table 2:** Precision and recall comparison of anomaly detection.

Anomaly	Precision					Recall				
	SFAD	OCSVM	MFAD	LSTM-NDT	SRAD	SFAD	OCSVM	MFAD	LSTM-NDT	SRAD
1	**0.7697**	0.6890	0.6371	0.4655	0.4516	**0.9750**	0.9417	0.6583	0.4500	0.4667
2	**0.9742**	0.9285	0.7381	0.4700	0.4868	0.9714	**1.0000**	0.7971	0.4029	0.5257
3	**0.9857**	0.9267	0.9378	0.9648	0.9425	0.9562	0.9608	**0.9724**	0.8848	0.9447
4	**0.9538**	0.9330	0.7994	0.8349	0.2527	0.9750	0.9540	0.7972	0.8428	0.2583
5	0.8727	0.8689	**0.8764**	0.6513	0.8534	**0.8962**	0.8923	**0.9000**	0.6538	0.8731
6	**0.9182**	0.9061	0.6174	0.8614	0.3971	0.8975	**0.9650**	0.6575	0.6525	0.4050
7	**0.8659**	0.8449	0.1798	0.1793	0.1291	**0.7100**	0.6992	0.1600	0.2600	0.4700

**Table 3:** F1-score and FPR comparison of anomaly detection.

Anomaly	F1-Score					FPR				
	SFAD	OCSVM	MFAD	LSTM-NDT	SRAD	SFAD	OCSVM	MFAD	LSTM-NDT	SRAD
1	**0.8603**	0.7958	0.6475	0.4576	0.4590	**0.0350**	0.0509	0.0450	0.1351	0.0679
2	**0.9728**	0.9643	0.7665	0.4338	0.5055	**0.0030**	0.0106	0.0388	0.1384	0.0760
3	**0.9708**	0.9434	0.9547	0.9231	0.9436	**0.0024**	0.0134	0.0113	0.0131	0.0101
4	**0.9643**	0.9424	0.7983	0.8388	0.2555	**0.0130**	0.0141	0.0550	0.4862	0.2101
5	0.8843	0.8805	**0.8880**	0.6526	0.8631	0.0224	0.0231	**0.0218**	0.4272	0.0258
6	0.9077	**0.9346**	0.6368	0.7425	0.4010	**0.0232**	0.0291	0.1186	0.5753	0.1790
7	**0.7802**	0.7631	0.1693	0.2122	0.2026	**0.0064**	0.0084	0.0426	0.1541	0.1850

**Table 4:** Evaluation indicators of different dictionaries in SFAD.

Anomaly	K-SVD-OMP-OCSVM				DCT-OMP-OCSVM				TD-OMP-OCSVM			
	P	R	F1	FPR	P	R	F1	FPR	P	R	F1	FPR
1	**0.7697**	**0.9750**	**0.8603**	**0.0350**	0.4944	0.7333	0.5906	0.0903	0.5645	0.5833	0.5738	0.0539
2	**0.9742**	**0.9714**	**0.9728**	**0.0030**	0.5961	0.7886	0.6789	0.0733	0.4365	0.4714	0.4533	0.0835
3	**0.9857**	**0.9562**	**0.9708**	**0.0024**	0.6410	0.9009	0.7490	0.0887	0.7399	0.8848	0.8059	0.0547
4	0.9538	0.9750	0.9643	0.0130	**0.9673**	**0.9972**	**0.9820**	**0.0092**	0.8207	0.8389	0.8297	0.0504
5	**0.8727**	**0.8962**	**0.8843**	**0.0224**	0.8202	0.8423	0.8311	0.0317	0.7821	0.8423	0.8111	0.0402
6	**0.9182**	**0.8975**	**0.9077**	**0.0232**	0.9028	0.8825	0.8925	0.0276	0.4680	0.4575	0.4627	0.1513
7	**0.8659**	0.7100	**0.7802**	**0.0064**	0.6571	**0.9200**	0.7667	0.0281	0.8415	0.6900	0.7582	0.0076

Note: P means precision, R means recall, and F1 means F1-score.

**Table 5:** MSL Data Information.

Attributes	Values
Total anomaly sequences	36
Point anomalies (% tot.)	19 (53%)
Contextual anomalies (% tot.)	17 (47%)
Unique telemetry channels	27
Telemetry values evaluated	66,709

**Table 6:** Comparison of Anomaly Detection Results on the MSL Dataset.

	Precision	Recall	F1-Score
SFAD	0.871	**0.750**	**0.806**
OCSVM	0.774	0.667	0.750
MFAD	0.815	0.611	0.698
LSTM-NDT	**0.926**	0.694	0.793
SRAD	0.714	0.417	0.527

## Data Availability

Not applicable.
